# Exposure to Spoken Communication During the COVID-19 Pandemic Among Children With Cochlear Implants

**DOI:** 10.1001/jamanetworkopen.2023.39042

**Published:** 2023-10-27

**Authors:** Emily Wener, Lindsay Booth, Hailey Bensky, Veeral Desai, Jaina Negandhi, Sharon L. Cushing, Blake C. Papsin, Karen A. Gordon

**Affiliations:** 1 Archie’s Cochlear Implant Laboratory, The Hospital for Sick Children, Toronto, Ontario, Canada; 2 Department of Otolaryngology–Head & Neck Surgery, University of Toronto, Toronto, Ontario, Canada; 3 Department of Otolaryngology, The Hospital for Sick Children, Toronto, Ontario, Canada; 4 Department of Communication Disorders, The Hospital for Sick Children, Toronto, Ontario, Canada

## Abstract

**Importance:**

School closures and other COVID-19–related restrictions could decrease children’s exposure to speech during important stages of development.

**Objective:**

To assess whether significant decreases in exposure to spoken communication found during the initial phase of the COVID-19 pandemic among children using cochlear implants are confirmed for a larger cohort of children and were sustained over the first years of the COVID-19 pandemic.

**Design, Setting, and Participants:**

This cohort study used datalogs collected from children with cochlear implants during clinical visits to a tertiary pediatric hospital in Toronto, Ontario, Canada, from January 1, 2018, to November 11, 2021. Children with severe to profound hearing loss using cochlear implants were studied because their devices monitored and cataloged levels and types of sounds during hourly use per day (datalogs) and because their hearing and spoken language development was particularly vulnerable to reduced sound exposure. Statistical analyses were conducted between January 2022 and August 2023.

**Main Outcomes and Measures:**

Daily hours of sound were captured by the cochlear implant datalogging system and categorized into 6 auditory scene categories, including speech and speech-in-noise. Time exposed to speech was calculated as the sum of daily hours in speech and daily hours in speech-in-noise. Residual hearing in the ear without an implant of children with unilateral cochlear implants was measured by pure tone audiometry. Mixed-model regression analyses revealed main effects with post hoc adjustment of 95% CIs using the Satterthwaite method.

**Results:**

Datalogs (n = 2746) from 262 children (137 with simultaneous bilateral cochlear implants [74 boys (54.0%); mean (SD) age, 5.8 (3.5 years)], 38 with sequential bilateral cochlear implants [24 boys (63.2%); mean (SD) age, 9.1 (4.2) years], and 87 with unilateral cochlear implants [40 boys (46.0%); mean (SD) age, 7.9 (4.6) years]) who were preschool aged (n = 103) and school aged (n = 159) before the COVID-19 pandemic were included in analyses. There was a slight increase in use among preschool-aged bilateral cochlear implant users through the pandemic (early pandemic, 1.4 h/d [95% CI, 0.3-2.5 h/d]; late pandemic, 2.3 h/d [95% CI, 0.6-4.0 h/d]) and little change in use among school-aged bilateral cochlear implant users (early pandemic, −0.6 h/d [95% CI, −1.1 to −0.05 h/d]; late pandemic, −0.3 h/d [95% CI, −0.9 to 0.4 h/d]). However, use decreased during the late pandemic period among school-aged children with unilateral cochlear implants (−1.8 h/d [95% CI,−3.0 to −0.6 h/d]), particularly among children with good residual hearing in the ear without an implant. Prior to the pandemic, children were exposed to speech for approximately 50% of the time they used their cochlear implants (preschool-aged children: bilateral cochlear implants, 46.6% [95% CI, 46.5%-47.2%] and unilateral cochlear implants, 52.1% [95% CI, 50.7%-53.5%]; school-aged children: bilateral cochlear implants, 47.6% [95% CI, 46.8%-48.4%] and unilateral cochlear implants, 51.0% [95% CI, 49.4%-52.6%]). School-aged children in both groups experienced significantly decreased speech exposure in the early pandemic period (bilateral cochlear implants, −12.1% [−14.6% to −9.4%]; unilateral cochlear implants, −15.5% [−20.4% to −10.7%]) and late pandemic periods (bilateral cochlear implants, −5.3% [−8.0% to −2.6%]; unilateral cochlear implants, −11.2% [−15.3% to −7.1%]) compared with the prepandemic baseline.

**Conclusions and Relevance:**

This cohort study using datalogs from children using cochlear implants suggests that a sustained reduction in children’s access to spoken communication was found during more than 2 years of COVID-19 pandemic-related lockdowns and school closures.

## Key Points

**Question:**

Did decreases in exposure to spoken communication, found in the early stages of the COVID-19 pandemic among children using cochlear implants, resolve as lockdowns became more intermittent in later pandemic stages?

**Findings:**

In this cohort study, sound environments cataloged using machine learning for cochlear implants were measured by 2746 datalogs for 262 children using cochlear implants before and during 2 years of COVID-19 lockdowns in Ontario, Canada. Due to school closures during lockdowns, school-aged children experienced significantly decreased exposure to spoken language, which has not recovered to the prepandemic baseline.

**Meaning:**

This study suggests that school closures due to COVID-19 lockdowns are associated with reduced exposure to spoken communications among children using cochlear implants during sensitive periods of development.

## Teaser

This cohort study assesses whether significant decreases in exposure to spoken communication found during the initial phase of the COVID-19 pandemic among children using cochlear implants are confirmed for a larger cohort of children and were sustained over the first years of the COVID-19 pandemic.

## Introduction

Over the course of the COVID-19 pandemic, children experienced COVID-19 infections^1,2^ as well as related public health measures. Restricted access to health care settings delayed medical diagnoses, including hearing loss identification,^3,4^ for children and exacerbated gaps in access to mental health care in this age group due to limited capacity^5^ and an increase in need.^6,7,8^ Such concerns relate, in part, to the social isolation of COVID-19 lockdowns.^9^ Social isolation may be particularly important to consider for children with hearing loss who already have reduced access to sound and are at risk for delayed language development.^10,11,12,13,14,15^

Children with severe to profound hearing loss can gain access to spoken communication through cochlear implants that translate sound into electrical pulses that stimulate the auditory pathways. Many children with cochlear implants gain age-appropriate spoken language.^16^ Outcomes improve when the delay to implantation is minimized,^11^ families have sufficient access to support and resources,^17^ the child engages frequently in conversational turn-taking,^18^ and the devices are used consistently.^12,19,20^ The latter aspect has been monitored through datalogging systems in cochlear implant equipment. These systems clock the number of daily hours the cochlear implant is used and categorize incoming sounds using a machine learning algorithm.^21^ Previous analyses of the datalogs from children’s cochlear implants reveal that daily use increases from infancy into ages at which children begin school^22,23^ and confirm that speech is the most common sound to which children are typically exposed.^24^

Given datalog sensitivity to auditory environments, these measures were used to quantify changes among children associated with early COVID-19 pandemic lockdowns (March-July 2020). Datalogs from a cohort of 45 children revealed that they consistently used their cochlear implants but experienced a significant reduction in exposure to spoken language.^24^ This finding was particular to school-aged children, who lost approximately 1 hour or 10% of time exposed to speech during the initial lockdowns relative to their prepandemic exposure. As the pandemic has extended well beyond the initial period in 2020, the objective of the present study was to assess whether initial findings of significant decreases in exposure to spoken communication during the initial phase of the COVID-19 pandemic among children with severe to profound hearing loss who are using cochlear implants to hear are confirmed in a larger cohort of children and whether the decrease found in the early stages of the pandemic resolved as lockdowns became more intermittent in later stages of the pandemic.

## Methods

**Figure 1.**
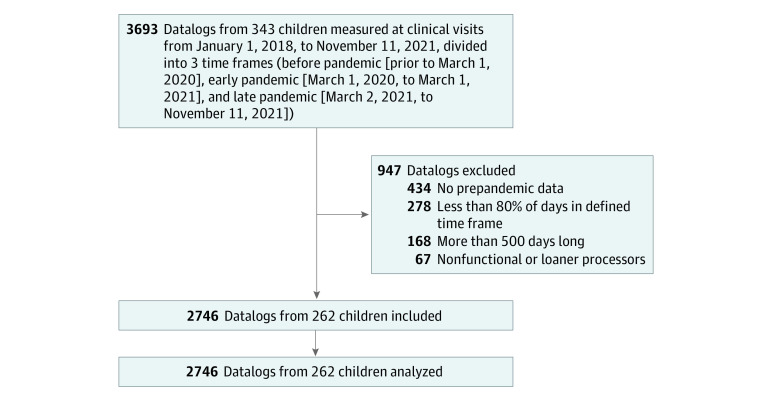
Flowchart of Data Inclusion and Exclusion

Written consent by parents, caregivers, or participants was obtained in this cohort study as approved by the Hospital for Sick Children’s Research Ethic Board, which adheres to the Tri-Council Policy on Ethical Conduct for Research Involving Humans. As shown in the flowchart (Figure 1), 3693 datalogs captured from the speech processors of 343 children with cochlear implants by connecting to the programming software (Cochlear Ltd) during clinical visits to a tertiary pediatric hospital in Toronto, Ontario, Canada, from January 1, 2018, to November 11, 2021, were identified for study. Inclusion and exclusion of data are shown as per the Strengthening the Reporting of Observational Studies in Epidemiology (STROBE) reporting guideline for cohort studies. Each datalog capture initiates a new period of collection. The data include the mean number of hours per day the device was worn in the period of data capture and hours per day of acoustic input, categorized by (1) intensity level (40-90 A-weighted dB in 10-dB bands) and (2) environment type (quiet, speech, speech-in-noise, music, noise, and other) determined by a validated automatic scene classifier.^25^

Datalogs were then classified into 3 pandemic-related time frames. Provincial lockdowns began in March 2020 and included closure of nonessential businesses and schools, restricted outpatient hospital visits, and restricted contact with individuals outside the home. School reopening occurred in stages beginning in September 2020 with full reopening of schools occurring in mid-February 2021, with subsequent disruptions according to case surges throughout 2021 into early 2022.^26^ Pandemic time frames were thus defined as (1) prepandemic (prior to March 1, 2020), (2) early pandemic (March 1, 2020, to March 1, 2021), and (3) late pandemic (March 2 to November 11, 2021). As shown in the flowchart (Figure 1), each datalog was assigned to a time frame if more than 80% of its days occurred within that period (278 datalogs excluded) and if datalogs were less than 500 days long (168 datalogs excluded). Datalogs from nonfunctional or loaner processors were also excluded (n = 67) as were data from children who had no prepandemic data (n = 434 datalogs excluded). This left 2746 datalogs from 262 children for inclusion in study analyses.

### Statistical Analysis

**Figure 2.**
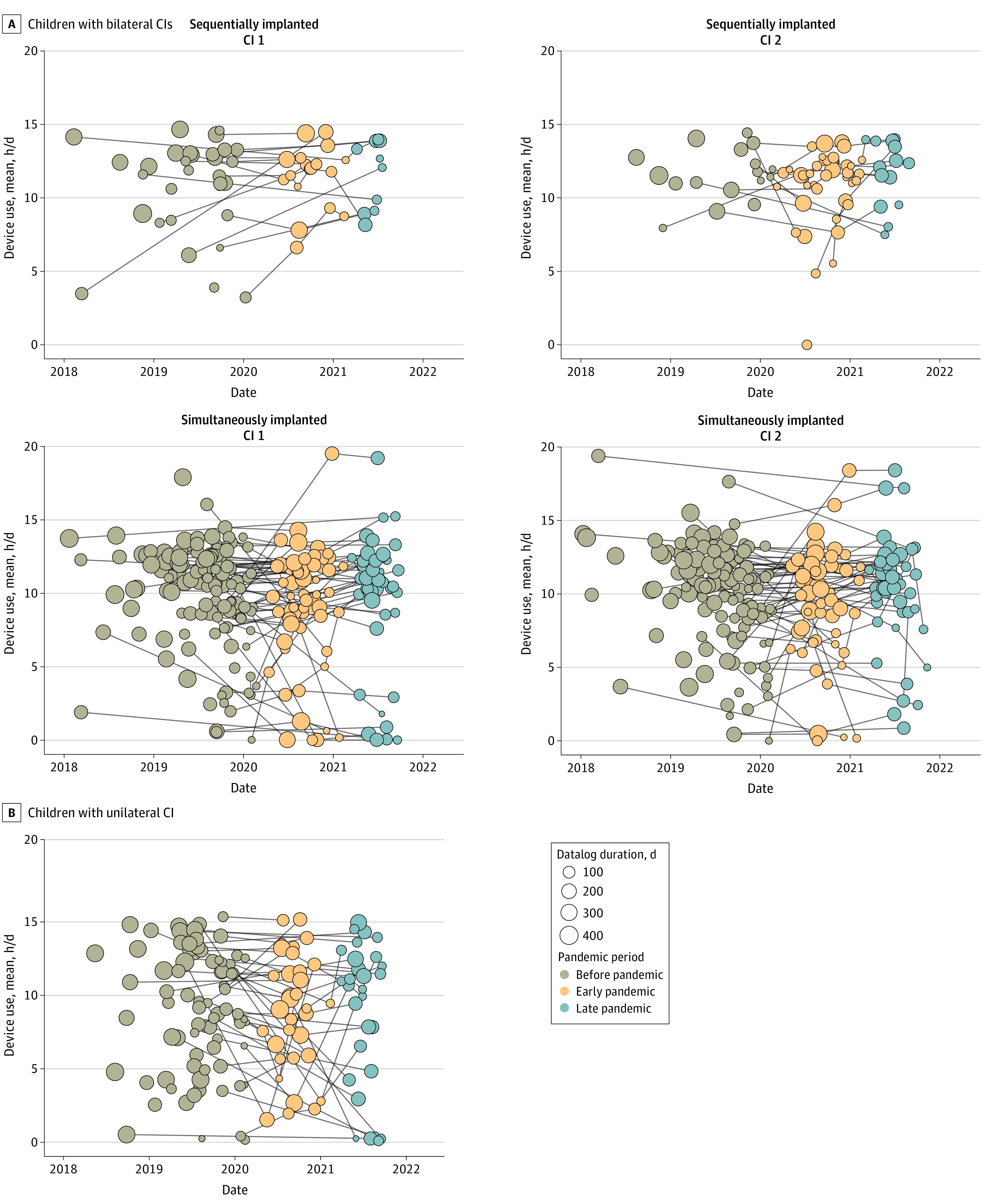
Datalogs Plotted by Median Date for Each Child, Reflecting 3 Time Frames for Analyses (Prepandemic, Early Pandemic, and Late Pandemic) Repeated measures are shown by lines connecting points. A total of 25 prepandemic datalogs with median dates prior to January 1, 2018, are not shown. Cochlear implant (CI) 1 = first CI received by children with sequential bilateral implants or right CI among children with simultaneous bilateral implants; CI 2 = second CI received by children or left CI among children with simultaneous bilateral implants.

**Figure 3.**
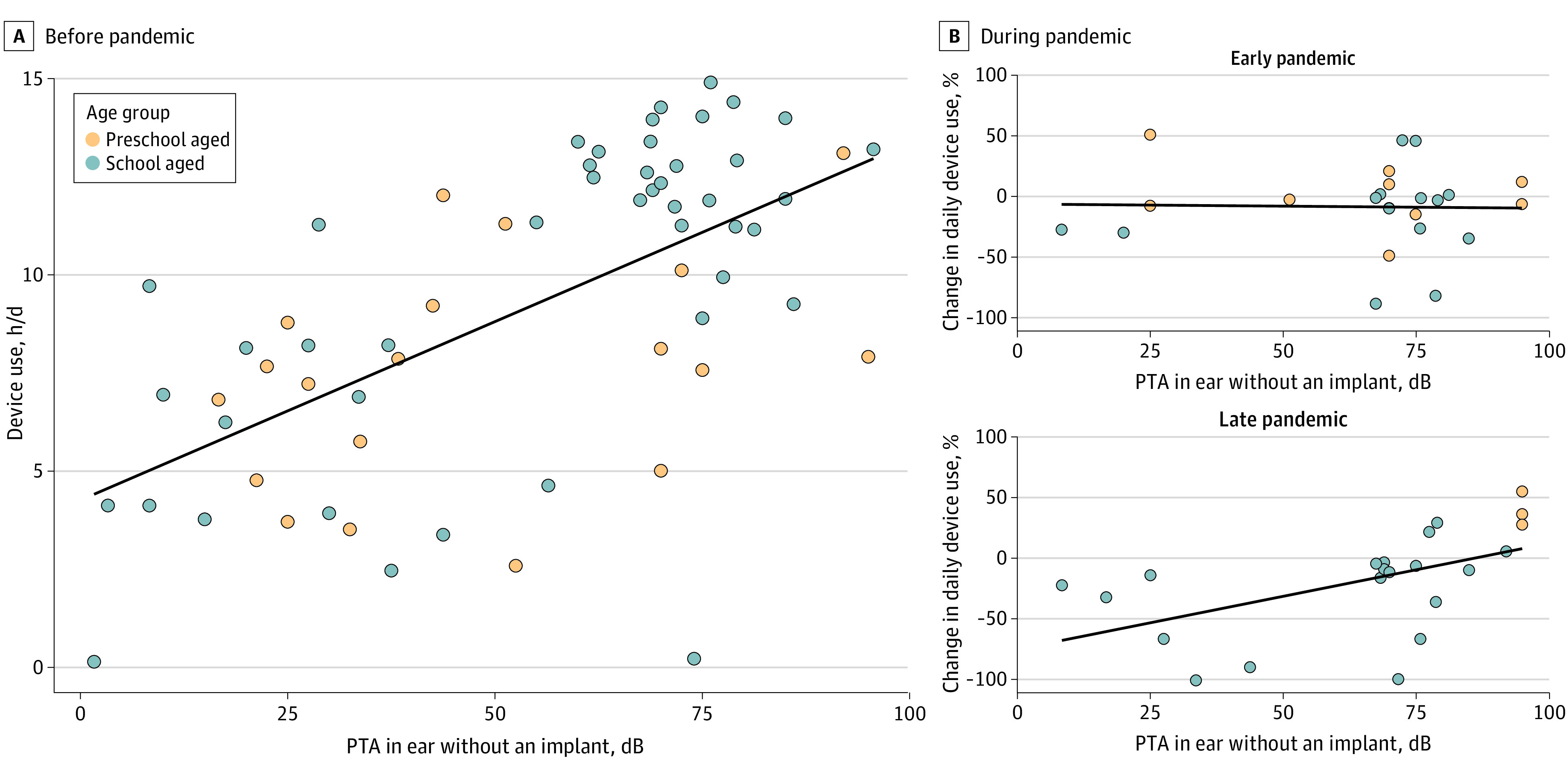
Unilateral Cochlear Implant Use Before the Pandemic Compared With Early and Late Pandemic Periods A, Device use increased with increasing pure tone threshold average (PTA) in the contralateral ear without an implant (mean [SE], 0.09 [0.02] hours per increase in pure tone thresholds; *P* < .001). B, Reductions in cochlear implant use during the late pandemic period relative to prepandemic levels increased for children with better hearing (lower PTA) (early pandemic, −0.04 hours per increase in decibel pure tone thresholds [95% CI, −0.7 to 0.6 h/dB pure tone thresholds]; late pandemic, 0.11 hours per increase in decibel pure tone thresholds [95% CI, 0.05-0.2 h/dB]).

Statistical analyses were conducted between January 2022 and August 2023. Datalog measures from both ears were included for children with bilateral cochlear implants. Linear mixed-effects regression analyses were conducted using the lmer4 package (Douglas Bates) and RStudio, version 1.4.1717 (RStudio Team; RStudio: Integrated Development Enivronment for R).^27,51^ Outcomes were hours of daily cochlear implant use, hours of daily speech exposure (data in speech and speech-in-noise categories), and percentage of speech exposure relative to daily cochlear implant use. Data from children with bilateral cochlear implants and those with unilateral cochlear implants were analyzed separately. Fixed effects were age group (preschool or school age) or age at datalog capture and pandemic period (prepandemic, early pandemic, or late pandemic). The ear with the cochlear implant (cochlear implant 1, right ear; cochlear implant 2, left ear) and group (simultaneously or sequentially implanted) were used as additional fixed effects in analyses of children with bilateral cochlear implants. Residual hearing in the ear without the implant, measured by pure tone audiometry, was used as an additional fixed effect in analyses of children with unilateral cochlear implants. A random intercept for each participant was included in all models. The mean values and 95% CIs estimated from the model and adjusted with the Satterthwaite method are reported in the text and Table 1 and Table 2. Figure 2 and Figure 3 include data from individual participants. All *P* values were from 2-tailed tests, and results were deemed statistically significant at *P* < .05.

**Table 1.** Demographic Characteristics of Participants

Characteristic	Simultaneous bilateral cochlear implants (n = 137)	Sequential bilateral cochlear implants (n = 38)	Unilateral cochlear implant (n = 87)
Age at cochlear implant, mean (SD), y	3.0 (0.2)	Cochlear implant 1: 4.6 (0.6)	5.3 (0.4)
		Cochlear implant 2: 7.7 (0.7)	
Male to female ratio	74:63	24:14	40:47
Age at prepandemic datalog, mean (SD), y	5.8 (3.5)	9.1 (4.2)	7.9 (4.6)
Preschool aged (<5 y) prepandemic, No.	68	6	29
School aged (≥5 y) prepandemic, No.	69	32	58
Ear that received cochlear implant 1 (left:right)	NA	16:22	40:47
PTA of ear without an implant, mean (SD), dB	NA	NA	53.3 (26.1)
PTA of ear with an implant, mean (SD), dB			
Right	97.2 (13.5)	Cochlear implant 1: 89.2 (16.7)	85.8 (18.9)
Left	96.5 (13.6)	Cochlear implant 2: 87.9 (20.0)	

Abbreviations: NA, not applicable; PTA, pure tone thresholds (as defined in Figure 3) and used in the text.

**Table 2.** Exposure to Speech (Speech-in-Noise Plus Speech Alone)

Characteristic	Preschool aged		School aged	
	Bilateral cochlear implants	Unilateral cochlear implant	Bilateral cochlear implants	Unilateral cochlear implant
Prepandemic				
Exposure, h/d (95% CI)	4.1 (3.7 to 4.4)	4.2 (3.8 to 4.7)	5.4 (5.1 to 5.7)	5.0 (4.6 to 5.3)
Percentage of daily cochlear implant use (95% CI)	46.6 (46.5 to 47.2)	52.1 (50.7 to 53.5)	47.6 (46.8 to 48.4)	51.0 (49.4 to 52.6)
**Pandemic changes**				
Early pandemic				
Exposure, h/d (95% CI)	0.7 (0.4 to 1.1)^a^	−0.6 (−1.6 to 0.5)	−1.6 (−2.0 to −1.2)^a^	−2.4 (−3.2 to −1.7)^a^
Percentage of daily cochlear implant use (95% CI)	6.2 (3.7 to 8.7)^a^	−5.1 (−11.6 to 1.5)	−12.1 (−14.6 to −9.4)^a^	−15.5 (−20.4 to −10.7)^a^
Late pandemic				
Exposure, h/d (95% CI)	1.6 (1.2 to 2.1)^a^	1.0 (−1.0 to 3.1)	−1.1 (−1.5 to −0.7)^a^	−2.1 (−2.7 to −2.0)^a^
Percentage of daily cochlear implant use (95% CI)	8.6 (5.6 to 11.6)^a^	−0.6 (−12.4 to 13.6)	−5.3 (−8.0 to −2.6)^a^	−11.2 (−15.3 to −7.1)^a^

^a^ Significant change.

## Results

The demographic characteristics of the 262 children (137 with simultaneous bilateral cochlear implants [74 boys (54.0%); mean (SD) age, 5.8 (3.5 years)], 38 with sequential bilateral cochlear implants [24 boys (63.2%); mean (SD) age, 9.1 (4.2) years], and 87 with unilateral cochlear implants [40 boys (46.0%); mean (SD) age, 7.9 (4.6) years]) included in this study are detailed in Table 1, and the median dates of their datalogs that met inclusion criteria are plotted in Figure 2. Datalog capture duration was not significantly different between time periods (prepandemic, 181.6 days [95% CI, 163.7-193.5 days]; early pandemic, 155.1 days [95% CI, 136.7-169.5 days]; late pandemic, 124.4 days [95% CI, 111.2-137.7 days]. Repeated measures from individual children are shown in Figure 2.

### Consistent Device Use During the Pandemic Among Preschool-Aged and School-Aged Children

Mean daily hours of cochlear implant use among children with bilateral cochlear implants were similar for children who received bilateral cochlear implants simultaneously (9.2 h/d [95% CI, 9.0-9.4 h/d]) and sequentially (10.4 h/d [95% CI, 10.1-10.7 h/d]), and there was no significant difference in daily cochlear implant use between implanted ears in either children with simultaneous bilateral cochlear implants (difference, 0.04 h/d [95% CI, −0.2 to 0.2 h/d]) or those with sequential bilateral cochlear implants (difference, 0.04 h/d [95% CI,−0.2 to 0.2 h/d]). Before the pandemic, school-aged children used their cochlear implants for slightly longer durations than preschool-aged children in both the bilateral (difference, 2.0 h/d [95% CI, 1.6-2.5 h/d]) and unilateral cochlear implant groups (difference, 1.8 h/d [95% CI, 0.9-2.7 h/d]). Cochlear implant use among those with unilateral cochlear implants was the same for children with left cochlear implants (8.9 h/d [95% CI, 7.8-9.9 h/d]) or right cochlear implants (8.3 h/d [95% CI, 7.1-9.4 h/d]).

Daily cochlear implant use among children with bilateral cochlear implants remained fairly stable over the course of the pandemic. There was a slight increase in use among the preschool-aged children with bilateral cochlear implants through the pandemic (early pandemic, 1.4 h/d [95% CI, 0.3-2.5 h/d]; late pandemic, 2.3 h/d [95% CI, 0.6-4.0 h/d]) and little change among school-aged children with bilateral cochlear implants (early pandemic, −0.6 h/d [95% CI, −1.1 to −0.05 h/d]; late pandemic, −0.3 h/d [95% CI, −0.9 to 0.4 h/d]). Given reduced initial prepandemic cochlear implant use among the preschool-age cohort, this meant they increased their bilateral cochlear implant use by 33.5% (95% CI, 15.8%-51.2%) in the early pandemic period and by 64.0% (95% CI, 32.1%-96.0%) in the late pandemic period. Conversely, school-aged children with unilateral cochlear implants showed decreases in cochlear implant use during the late pandemic period (−1.8 h/d [95% CI,−3.0 to −0.6 h/d] and −29.3% [95% CI,−47.8% to −10.9%] of prepandemic use).

Duration of cochlear implant use among children with unilateral cochlear implants was associated with residual hearing in the ear without the implant (0.1 h/dB [95% CI, 0.05-0.1 h/dB] pure tone threshold). As shown in Figure 3A, children with better hearing (lower pure tone thresholds) used their cochlear implants for less time daily before the pandemic. Reductions in cochlear implant use among the school-aged group were also associated with residual hearing in the late pandemic period. As plotted in Figure 3B, reductions were not associated with residual hearing in the early pandemic (−0.04 h/dB [95% CI,−0.7 to 0.6 h/dB] pure tone thresholds) but, in the late pandemic, were greater among children with better hearing in the ear without an implant (0.1 h/dB [95% CI, 0.05-0.2 h/dB] pure tone thresholds).

### Reduced Exposure to Speech During the Pandemic

Before the pandemic, the time exposed to speech (sum of speech alone and speech-in-noise) was longer among the school-aged children than the preschool-aged children by 1.4 h/d (95% CI, 0.9-1.8 h/d) with bilateral cochlear implants and by 0.7 h/d (95% CI, 0.2-1.2 h/d) with unilateral cochlear implants, consistent with longer daily cochlear implant use in the older group as reported. Children in both age groups with bilateral or unilateral cochlear implants were exposed to speech for approximately 50% of the time they used their cochlear implants (preschool-aged children: bilateral cochlear implants, 46.6% [95% CI, 46.5%-47.2%] and unilateral cochlear implants, 52.1% [95% CI, 50.7%-53.5%]; school-aged children: bilateral cochlear implants, 47.6% [95% CI, 46.8%-48.4%] and unilateral cochlear implants, 51.0% [95% CI, 49.4%-52.6%]) (Table 2). Data in eTable 1 and eTable 2 in Supplement 1 reveal that this exposure was made up of approximately 30% speech-in-noise and approximately 20% speech alone (in quiet).

Speech exposure changed during the pandemic from prepandemic levels with increases or no changes among preschool-aged children but clear reductions among school-aged children. As detailed in Table 2, preschool-aged children with bilateral cochlear implants had a significant increase in time exposed to speech in the early pandemic (0.7 h/d [95% CI, 0.4-1.1 h/d] and 6.2% [95% CI, 3.7%-8.7%]) and late pandemic (1.6 h/d [95% CI, 1.2-2.1 h/d] and 8.6% [95% CI, 5.6%-11.6%]). No significant changes were found for preschool-aged children with unilateral cochlear implants for either hours or percentage exposure to speech in either the early pandemic (−0.6 h/d [95% CI, −1.6 to 0.5 h/d] and −5.1% [95% CI, −11.6% to 1.5%]) or late pandemic (1.0 h/d [95% CI, −1.0 to 3.1 h/d] and −0.6% [95% CI, −12.4% to 13.6%]). These findings were similar for categories of speech-in-noise and speech alone (eTable 1 and eTable 2 in Supplement 1).

School-aged children showed reductions in exposure to speech of approximately 1 to 2 hours, which corresponded to losing approximately 10% to 15% of speech input (Table 2; eTable 1 and eTable 2 in Supplement 1). Reductions in school-aged children with bilateral cochlear implants occurred in both pandemic periods (early pandemic, −1.6 h/d [95% CI, −2.0 to 1.2 h/d] and −12.1% [95% CI, −14.6% to −9.4%]; late pandemic, −1.1 h/d [95% CI, −1.5 to −0.7 h/d] and −5.3% [95% CI, −8.0% to −2.6%]). Similar decreases in exposure to speech during the pandemic were found among school-aged children with unilateral cochlear implants (early pandemic, −2.4 h/d [95% CI, −3.2 to −1.7 h/d] and −15.5% [95% CI, −20.4% to −10.7%]; late pandemic, −2.1 h/d [95% CI, −2.7 to −2.0 h/d] and −11.2% [95% CI, −15.3% to −7.1%]). Analyses of speech-in-noise (eTable 1 in Supplement 1) were consistent, revealing increased exposure among preschool-aged children and reduced exposure among school-aged children during the pandemic. Changes in speech alone during the pandemic (eTable 2 in Supplement 1) also showed increased exposure among preschool-aged children and reductions among school-aged children with unilateral cochlear implants. School-aged children with bilateral cochlear implants showed little change in their exposure to speech alone during the pandemic.

## Discussion

Results of this study suggested that children with severe to profound sensorineural hearing loss who had bilateral cochlear implants used their devices as consistently during the COVID-19 pandemic as they did before the pandemic but that daily cochlear implant use was more variable among children with unilateral cochlear implants both before and during the pandemic. Before the pandemic, children using cochlear implants were exposed to speech for approximately 50% of the time they used their devices, but this amount decreased significantly during the pandemic among school-aged children, likely reflecting the main school closures occurring in our province over this 2-year period.

Children with bilateral cochlear implants do not have access to most sounds without their devices, which likely explains their reliance on and use of their cochlear implants for most of their waking hours.^28^ Device use was similar for children receiving bilateral cochlear implants simultaneously and those receiving bilateral cochlear implants sequentially, as shown previously in a similar cohort,^22^ although others have reported decreased use of the second cochlear implant when there were long interimplant delays between sequential surgeries.^29,30,31^ Daily cochlear implant use was longer for school-aged children than preschool-aged children, as previously reported,^24^ and has been linked to increasing daily hours of time awake from infancy to later childhood.^22^ This finding can also explain the increase in bilateral cochlear implant use among the younger group over the time of data collection.

More variable device use among children with unilateral cochlear implants was associated with residual hearing in the ear without the implant, suggesting that these children were less reliant on their cochlear implants even before the pandemic. This finding is consistent with reports of reduced hearing aid use among children with mild unilateral hearing loss.^32,33^ The pandemic appeared to exacerbate the more limited use of unilateral cochlear implants among school-aged children, particularly for children with better hearing in the ear without the implant. We speculate that the virtual environment, unlike the typical classroom or playground, was relatively quiet, thus requiring little bilateral or spatial hearing. It is possible that this change allowed school-aged children with good residual hearing in the ear without the implant to function without their cochlear implants. It is also likely that older children had sufficient autonomy to choose not to wear their cochlear implants. Reduced cochlear implant use among the school-aged children with unilateral implants appears to be specific to the pandemic, as prepandemic data from prior cohorts of children with single-sided deafness (normal residual hearing in the ear without the implant) indicated that they consistently used their cochlear implants over time.^21,34^

The reduced exposure to speech among school-aged children during the pandemic found in this study confirms prior results from a smaller cohort during initial pandemic lockdowns^24^ and shows that the pandemic’s association with exposure to speech was sustained over years. The datalogs provide a consistent quantification of sound environment changes resulting from pandemic-associated restrictions. Speech exposure reduction occurred among school-aged children and not younger preschool-aged children, reflecting the impact of school closures and increased virtual classroom teaching. Many of the young children were not in a regular daycare program or preschool during the study period, so their daily schedules might not have been changed by school closures.^35^ The finding of increased speech exposure among young children with bilateral cochlear implants over this time could also be due to spending more time with caregivers who were at home more frequently during the pandemic.

One important aspect of the speech logged by the cochlear implants is that the speakers are not known and the speech can come from in-person speakers or from electronic media.^36^ Several studies have highlighted increased screen time among children over the course of the pandemic for both educational and recreational purposes.^37,38,39,40,41^ Thus, school-aged children using cochlear implants probably experienced even fewer in-person conversations than the decreases in speech that were quantified in this study. Screen time has been linked to significant decreases in language development^42,43^; this association is exacerbated among children with hearing loss.^9,44^ Moreover, in-person speakers are typically engaged in conversation, whereas passive exposure to speech from electronic media could have a lesser association with promoting speech and language development.^45^

The decreased access to speech in childhood shown here could have developmental costs,^46,47^ including long-term associations of speech and language disorders such as reactive temperaments, hyperactive behaviors, and social withdrawal.^48,49,50^ Thus, the present findings suggest significant developmental risks of the COVID-19 pandemic among school-aged children. Although the cohort were children using cochlear implants, their peers with typical hearing were likely experiencing similar reductions in exposure to speech during the pandemic, with related developmental risks.

### Limitations

This study identifies the association of the pandemic with speech exposure among a large cohort of children using cochlear implants, but it is not without limitations. First, datalog time intervals were not uniform in length or dates across children. With multiple province-wide lockdowns, regularly scheduled appointments were disrupted, leading to prolonged intervals between appointments and datalog collection. The following criteria were applied to account for this heterogeneity: (1) datalogs of more than 500 days were excluded, and (2) if more than 80% of a given datalog did not occur within a given time period, it was excluded. Second, restrictions to in-person visits when datalogs were collected may have created selection bias. Third, the sound categorization algorithm was not able to discern a child’s own speech from that of another person or to identify speech coming from electronic media. These data may become available in the future, providing an opportunity to quantify conversational turns, to determine when these turns are restricted or facilitated, and to assess the associations of the home environment, including impacts of social determinants of health. Data collection in the present study ended in late 2021. Since then, pandemic-related restrictions have eased and the protocols for the 2022-2023 academic school year were similar to those before the pandemic, which will allow assessment of returns to prepandemic speech exposure. Longer-term follow-up will be needed to explore developmental effects of reduced speech exposure during the pandemic.

## Conclusions

This cohort study confirms earlier findings of reduced exposure to speech among school-aged children using cochlear implants at initial stages of the COVID-19 pandemic and demonstrates a sustained decrease over the following 2 years. The findings likely reflect experiences of many children through the pandemic and highlight the need for monitoring to prevent potential developmental sequelae of decreased speech exposure.

## Notes

Supplement 1. **eTable 1.** Mean (95% Confidence Interval) Exposure to Speech in Noise **eTable 2.** Mean (95% Confidence Interval) Exposure to Speech Alone

Supplement 2.
Data Sharing Statement
