# Supplementary material for: Exposure to Spoken Communication During the COVID-19 Pandemic Among Children With Cochlear Implants
Source: JAMA Netw Open. 2023 Oct 27;6(10):e2339042. doi: 10.1001/jamanetworkopen.2023.39042 (PMC10611997; doi:10.1001/jamanetworkopen.2023.39042)
Supplement: Supplement 2. — Data Sharing Statement [file jamanetwopen-e2339042-s002.pdf]

Data Sharing Statement

Wener. Exposure to Spoken Communication During the COVID-19 Pandemic Among Children With Cochlear Implants. *JAMA Netw Open*. Published October 23, 2023.
doi:10.1001/jamanetworkopen.2023.39042

Data
Data available: Yes
Data types: Deidentified participant data
How to access data: Deidentified data can be made available upon request with proper agreements in place.
When available: With publication

Supporting Documents
Document types: None

Additional Information
Who can access the data: researchers whose proposed use of the data has been approved
Types of analyses: For specific purposes such as systematic reviews.
Mechanisms of data availability: with a signed data access agreement
